# Evidence of G-Protein-Coupled Receptors (GPCR) in the Parasitic Protozoa *Plasmodium falciparum*—Sensing the Host Environment and Coupling within Its Molecular Signaling Toolkit

**DOI:** 10.3390/ijms222212381

**Published:** 2021-11-17

**Authors:** Pedro H. S. Pereira, Celia R. S. Garcia

**Affiliations:** Departamento de Análises Clínicas e Toxicológicas, Faculdade de Ciências Farmacêuticas, Universidade de São Paulo—USP, São Paulo 05508-900, Brazil; pedroscarpelli2@gmail.com

**Keywords:** *Plasmodium falciparum*, cell signaling, GPCRs, synchronization, calcium

## Abstract

Throughout evolution, the need for single-celled organisms to associate and form a single cluster of cells has had several evolutionary advantages. In complex, multicellular organisms, each tissue or organ has a specialty and function that make life together possible, and the organism as a whole needs to act in balance and adapt to changes in the environment. Sensory organs are essential for connecting external stimuli into a biological response, through the senses: sight, smell, taste, hearing, and touch. The G-protein-coupled receptors (GPCRs) are responsible for many of these senses and therefore play a key role in the perception of the cells’ external environment, enabling interaction and coordinated development between each cell of a multicellular organism. The malaria-causing protozoan parasite, *Plasmodium falciparum*, has a complex life cycle that is extremely dependent on a finely regulated cellular signaling machinery. In this review, we summarize strong evidence and the main candidates of GPCRs in protozoan parasites. Interestingly, one of these GPCRs is a sensor for K^+^ shift in *Plasmodium falciparum*, PfSR25. Studying this family of proteins in *P. falciparum* could have a significant impact, both on understanding the history of the evolution of GPCRs and on finding new targets for antimalarials.

## 1. An Overview of GPCRs in Multicellular Eukaryotes

G-protein-coupled receptors (GPCRs) have a common core of seven successive transmembrane domains (7TM) linked by three extra and intracellular loops, conserved among eukaryotes [1]. Half of the receptors of this family identified in humans have a sensory function including smell and taste [2]. The role of these receptors is to translate extracellular signals into intracellular signals caused by stimuli that can be from molecules of the most varied physicochemical characteristics (from ions to proteins). Some 40% of all drugs found on the pharmaceutical market have a GPCR family member as a target, which reflects the importance of these proteins in cellular functions [3,4].

When an activated molecule, or agonist, binds to a GPCR on the extracellular side, causing a conformational change mainly of the transmembrane helix 6 of the receptor, the signal is transmitted to the heterotrimeric G protein. These receptors do not act in a bimodal manner, and the signal can be activated at several intensities that result in several different signal results [5,6]. GPCRs do not act exclusively through the G protein: they can act in a G-protein-independent pathway; activate ion channels, transcription factors, arrestins and kinases [7]. Some GPCRs have constitutive activity causing constant activation of G proteins that can be modulated by the action of several molecules [8]. One GPCR activation is not exclusively related to one agonist. There is increasing interest in the so-called biased ligands: molecules that activate one pathway at the expense of other pathways [9,10]. GPCRs ligands are mainly agonists, but there are also antagonists and inverse agonists [11].

Gα, Gβ and Gγ are the subunits of heterotrimeric G proteins and in humans the sixteen genes for Gα proteins have been classified into four families by sequence similarity and their functions: Gs, Gi/o, Gq/11 and G12/13 [12,13]. Heterotrimeric G proteins stay bound to a GDP molecule in an inactive state until activation of a GPCR allows the GDP to dissociate. An activated GPCR-bound GDP free Gα subunit binds to soluble GTP, since its cytoplasmatic concentration is high, causing a conformational change that dissociates the heterotrimeric G protein into free Gα and Gβγ [14,15]. The free GTP-bound Gα subunit can activate several effectors, such as guanylate/adenylate cyclases, phosphodiesterases, phospholipases, and guanine exchange factors (GEFs) [16]. Free Gβγ also has a role in signal transduction: to recruit GRKs to the receptor, regulate ion channels, adenylate cyclases, phospholipase C and kinases [17]. Due to the intrinsic GTPasic activity of Gα, GTP is hydrolyzed, causing reassociation of Gα and Gβγ abolishing the response and completing the cycle of activation [16]. GTPase activation proteins (GAP) can accelerate the signal termination and contribute to the reassociation of heterotrimeric G proteins. An active GPCR is able to associate with another G protein complex and initiate another cycle of signaling as long as it remains in the active state. Therefore, a GPCR desensitization process occurs in order to prevent the receptors from remaining active after the first propagation of the signal. This process happens by two steps: the action of G-protein-coupled receptor kinases (GRKs) that phosphorylate the GPCR C-tail and internalization for degradation or recycling of GPCRs by β-arrestin.

GRKs physically bind to the GPCRs by phosphorylating the C-terminal end of the receptor when the heterotrimeric G protein is absent. Phosphorylated GPCRs have lower activity, but the activation of G proteins does not cease [18]. GRKs can also interact with free Gβγ subunits, disrupting the signaling caused by these subunits. Besides participating in the desensitization of GPCRs, GRKs have other substrates [18]. β-arrestins are responsible for signal termination through a process known as GPCR desensitization. Both Gα and β-arrestin compete for the internal cavity of a GPCR cytoplasmatic side [19,20,21]. The phosphorylated C-terminal of an activated GPCR increases the affinity of β-arrestins, strongly binding to this region and favoring the process of desensitization [22,23]. β-arrestins have a C-terminal domain that is exposed after binding to a GPCR and can recruit clathrin and adaptor protein 2 (AP2) to start the internalization. In other words, β-arrestin not only physically blocks the binding of G proteins in an activated GPCR, but also promotes clathrin-dependent internalization, interrupting stimulus and signal transduction [21,24].

## 2. Molecular Machinery for Cellular Signaling in *Plasmodium* Genus

**Figure 1:**
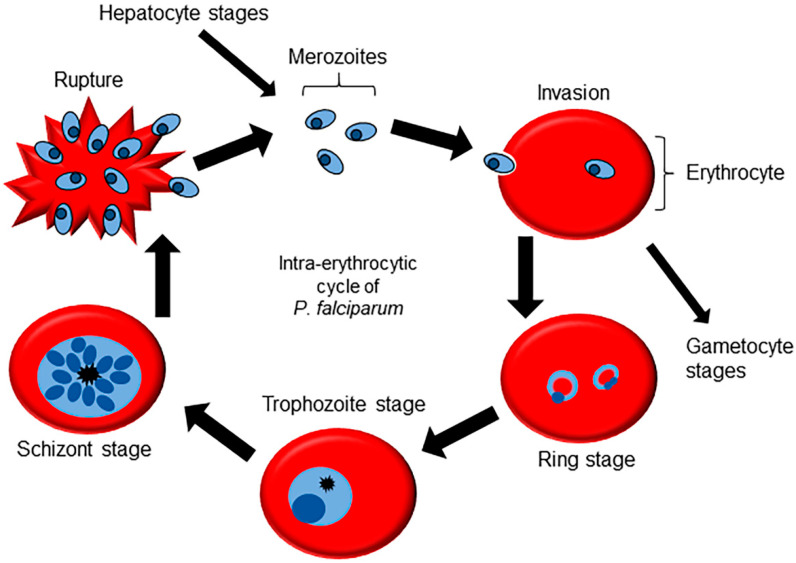
Schematic figure of the intraerythrocytic cycle of *P. falciparum*. Merozoites from the liver stages are released into the bloodstream and invade the erythrocytes, differentiating into rings. The ring stage grows in volume taking advantage of the abundance of nutrients until it becomes a trophozoite, at which stage the metabolic activity is high and DNA replication begins. The development is completed in the schizont stage, where it is possible to notice several nuclei that will later segment into new merozoites. The mature merozoites rupture the RBC and are released into the bloodstream ready to invade new cells and complete the cycle. A small portion of the merozoites that invade the cells differentiate into gametocytes, which, over 14 days, complete the development and are able to infect mosquitoes.

The *P. falciparum* life cycle starts with the bite of a female *Anopheles* mosquito, injecting saliva containing sporozoites into the bloodstream [25]. The sporozoites migrate to the liver invading hepatocytes, where asexual replication occurs, resulting in the formation of a large number of merozoites. The merozoites are released back into the bloodstream and are able to infect red blood cells, initiating the intraerythrocytic cycle [26,27]. After invasion, the merozoites pass through three morphologically different stages in a defined interval of 48 h (Figure 1): ring stage, where metabolism is low; trophozoite stage, in which there are protein and nucleic acid synthesis peaks while increasing cell volume; and schizont stages, where cell division occurs for the formation of new merozoites [28]. The schizonts rupture the host cell to release the merozoites into the bloodstream, continuing the erythrocytic cycle and giving rise to the main symptoms of malaria infection [29]. A small percentage of the parasites are able to break out of this cycle and engage in a process of differentiation into female and male gametocytes, which represent the infective forms to the mosquito vector. When the mosquito has a blood meal from an infected host, the gametocytes mature into gametes in the gut of the vector, which is followed by fertilization and generation of the zygote [30]. The zygote crosses the intestinal epithelium and differentiate into an oocyst. The oocysts go through successive cycles of cell division, until they rupture and release sporozoites, which migrate to the salivary gland of the mosquito, finishing the cycle [31].

The parasites of the *Plasmodium* genus have a complex cell signaling system capable of regulating all stages of parasite development [32]. Among the secondary messengers involved in this process are Ca^2+^ ions and cAMP, both of which are also essential for GPCR signaling in mammals. The process of in vivo parasite maturation occurs in a synchronous way, meaning that schizonts rupture the red blood cells and invade new cells at 48 h intervals, for *Plasmodium falciparum*. It has been shown that melatonin produced by the host is at least partially responsible for this synchrony [33,34,35]. Melatonin has been shown to have a role in regulating gene expression including the ubiquitin proteasome system (UPS) and the transcription factors PfNFYB and PfMORC [36,37,38,39,40].

*P. falciparum* protein kinase 7 (PfPK7) is an orphan kinase found only in *Plasmodium* parasites. The C-terminal of PfPK7 bears homology to the mitogen-activated protein kinase kinase (MAPKK), and the N-terminal bears similarity to protein kinase A (PKA) found in fungi [41]. Disruption of the pfpk7 gene is not lethal in vitro, but results in slow growth [42]. Interestingly, the PfPK7 knockout strain (PfPK7-) do not respond to melatonin: after treatment with the hormone, the proportion of asexual stages do not change, the increase in cytosolic Ca^2+^ is lower when compared to the wild-type strain, and there is no modulation of UPS genes [43]. The phosphoproteome of PfPK7- in schizonts resulted in the identification of 1047 proteins differently phosphorylated when compared to wild type Pf3D7 parasites [44]. In addition to PfPK7, the eukaryotic initiation factor kinase of *P. falciparum* (PfeIk1) is also involved in this signaling pathway, and its knockout is able to abolish the effect of melatonin on parasite synchronization [45].

The melatonin signal transduction in *P. falciparum* depends on the activation of PLC/IP3 pathway, including the activation of phospholipase C, which induces the formation of inositol triphosphate (IP3), leading to an increase in cytosolic Ca^2+^ concentration [46,47,48]. All attempts to elucidate the IP3 receptor in apicomplexan parasites have failed and no plausible candidate has been identified using bioinformatics and homology to other organisms [49]. This is not surprising considering the phylogenetic distance between the parasite and the mammalian counterparts. Although all attempts to find IP3R using bioinformatics analyses have failed, a recent study has merged affinity chromatography with bioinformatics meta-analyses to identify possible membrane proteins capable of binding to IP3. Among the results, the multidrug resistance transporter 1 (PfMDR1—PF3D7_0523000) stands out, a transporter widely studied due to its important role in resistance to several antimalarials located in the membrane of the digestive vacuole, essential for the survival of the parasite and capable of binding to IP3 [50].

Extracellular ATP is one of the most ubiquitous signalers, present in different biological models with varied functions. *P. falciparum* parasites sense extracellular ATP, causing a signaling cascade that results in an increase in cytoplasmic Ca^2+^ in trophozoites and schizonts [51]. In the in vitro culture of *P. falciparum*, the addition of apyrase, an enzyme that degrades nucleotides, or of purinergic receptor antagonists, dramatically reduces the rate of invasion of new RBCs [52,53]. These results opened up an area of research as they constitute strong evidence for the relevance of the role of ATP-mediated signaling in the processes of RBC invasion by the parasite.

Cyclic nucleotide (cAMP and cGMP) formation occurs through the cyclases (adenylate cyclase (AC) or guanylate cyclase (GC)) on the triphosphate forms of each nucleotide (ATP or GTP) forming a phosphodiester bond. The breaking of this phosphodiester bond by a cyclic nucleotide phosphodiesterase (PDE) causes disruption of the signal sent by cAMP and cGMP. Cyclic nucleotides stimulate the action of guanine exchange protein directly activated by cAMP (EPAC) and kinases such as PKA and PKG.

*Plasmodium* cyclases are structurally and functionally divergent from other organisms. *Plasmodium falciparum* ACα (PfACα) is insensitive to a known activator of AC in mammalian cells, forskolin. In addition, PfACα has a six transmembrane region that shares homology to voltage-dependent potassium channels [54,55]. PfACα knockout does not change parasite growth rate during the erythrocytic cycle, the survival during this stage, rate of gametocyte differentiation, mosquito stage growth or sporozoite formation. The infection of hepatocytes by sporozoites, on the other hand, show a reduced infection rate due to the incapability of apical proteins exocytosis [56]. Another cyclase, *Plasmodium falciparum* Acβ (PfACβ), is a human bicarbonate-sensitive soluble AC orthologous, essential for parasite survival and proliferation. There is a concentration-dependent increase in cytoplasmic Ca^2+^ and secretion of microneme proteins coupled to cAMP synthesis in merozoites. After pharmacological inhibition of PfACβ, this process is interrupted, highlighting the role of PfACβ in parasite invasion [57,58].

Thélu et al. (1994) identified Gα-like proteins in *P. falciparum* for the first-time, using extracts positive for Ras-like proteins during all stages of erythrocyte development. Furthermore, by using antibodies against the catalytic site common to all heterotrimeric G proteins a specific positive band was identified, but no band was observed when using an antibody against the C-terminal region of Gi and Gs [59]. Using radiolabeled ADP-ribosylation by cholera and pertussis toxins followed by Western blotting, a total of six bands with a molecular mass close to that expected for a G protein, between 34 and 54 kDa, were identified throughout the erythrocytic cycle, including gametocytes [60]. These toxins are known specific inhibitors of proteins of the Gαs and Gαi/o families. In addition, the same work detected a correlation between cholera toxin treatment and gametocyte formation, indicating a link between signaling and cell differentiation. Kaiser A. et al. (2015) described for the first time a non-canonical Ras-like G protein from *Plasmodium* (PfG—PF3D7_0313500) demonstrating GTP-binding capacity, GTPase activity, cytoplasmic localization, and a divergence from any known alpha subunit [61]. Furthermore, the possibility that *Plasmodium* uses the erythrocyte’s GPCR machinery cannot be excluded: it has been reported that the parasite uses endogenous β-adrenergic receptors and Gαs during the invasion process, and blocking them causes a reduction in parasitemia [62]. A comparative summary of GPCRs signaling pathway components between *H. sapiens* and *P. falciparum* can be found in Table 1.

## 3. In Silico Studies for the Identification of GPCR Candidates in *Plasmodium falciparum*

Madeira et al. (2008) performed a search in the *Plasmodium* genome aiming to identify GPCRs candidates in this organism. The approach for this search is indirect because the similarity of amino acid sequences between GPCRs is very small, even within the same species. For this purpose, all open reading frames of the genome were filtered for the presence of seven transmembrane domains using the TMHMM 2.0 algorithm, resulting in a total of sixty-six candidates. After this step, the candidates were filtered for several parameters, such as: conserved domains, similarity to other known proteins, signal sequences, presence of repeats, and finally were crossed with the possible GPCRs described by Inoue, Ikeda, and Shimizu, (2004) [63]. The analyses resulted in four promising candidate GPCRs, named PfSR1, PfSR10, PfSR12 and PfSR25 [64]. Due to filter restrictions several candidates were discarded, among them three that have an apicoplast targeting signal sequence PF3D7_1213500 (integral membrane protein GPR180, putative), PF3D7_1445800 (conserved *Plasmodium* membrane protein, unknown function) and PF3D7_1463900 (rhoptry neck protein 11, putative). A summary of the characteristics for each of the four putative GPCRs can be found in Table 2.

PfSR1 has 773 amino acids, and although the analyses performed by the SignalP 3.0 algorithm did not reveal the presence of a signal peptide, four other tools made the prediction of a plasma membrane localization signal sequence located at the N-terminal end (TMAP, TOPPRED2, TMPRED and HMMTOP) [64]. The N-terminal end is very long, with 508 amino acids, which brings this candidate closer to class C receptors, as predicted previously, using binary topology pattern methodology [63]. PfSR1 has a similarity with a region of CLPTM1 (cleft lip and palate transmembrane protein 1), whose function is not yet well established. This protein in humans and murines is located in the plasma membrane and is related to cell division, cancer, apoptosis, and other processes. The similarity comes from the multiple transmembrane regions present in both proteins. It is worth mentioning that the genus *Plasmodium* has an orthologous copy of CLPTM1 described in the genome, with no defined function. As for the similarity among the different *Plasmodium* species, *P. falciparum* and *P. reichenowi* are highly similar (93%), standing out from the other members of the genus, which share less than 50% similarity. Large-scale mutagenesis experiments indicated that deletion of the PfSR1 gene is not lethal, but causes a considerable fitness loss, leading to a 56% growth rate relative to the wild [65].

PfSR10 has 665 amino acids and a canonical plasma membrane localization signal peptide with a long N-terminal region of 381 amino acids, which resembles the size of hormone receptors such as follicle stimulating hormone and luteinizing hormone, both class A as classified using binary topology pattern methodology [63]. PfSR10 homologs can be found in all *Plasmodium* species, with a similarity greater than 70%. The lung seven transmembrane receptor domain (LUSTR) was identified due to the high homology of the transmembrane region, which is encountered in several orphan GPCRs. Tchoufack, N. et al. (2020) developed polyclonal antibodies using the N-terminal sequence of PfSR10 and genetically engineered parasites to express PfSR10-hemagglutinin tagged, which provided evidence for the subcellular localization of this protein: there is partial colocalization with endoplasmic reticulum markers, and presence in vesicular structures in intraerythrocytic stages and in gametocytes [67]. This localization matches the preliminary data described by Marapana et al. (2018) that describe SR10 as a molecular partner of the ER-resident signal peptide peptidase [68]. In contrast to the data presented by Nijila Tchoufack et al. (2020), high throughput mutagenesis experiments deposited in PlasmoDB [69] indicate that SR10 is not essential and does not cause significant changes in the growth of the parasites, both *P. falciparum* and *P. chabaudi*. As for the functional analysis, a knockout had a decrease in parasitemia by one third in the intraerythrocytic cycle and gametocyte production dropped by half, without any visible morphological change [67]. Subudhi et al. (2020) elegantly demonstrated that SR10 knockout causes the total duration of the intraerythrocytic cycle to decrease by 3 h, in addition to altering the rhythmic expression of several genes such as DNA replication, RNA processing, and UPS, highlighting the role of this protein in controlling the parasite cell cycle [70].

PfSR12 has a long N-terminal region of 231 amino acids out of 470. Like PfSR1, the homology of PfSR12 between *Plasmodium* species is quite divergent: the highest similarity found with Sr12 from *P. falciparum* is 62% in *P. chabaudi*. However, unlike the other putative receptors, there is a wide homology with another protein found in mammals, identified by PFAM, INTERPRO and PANTHER databases: both transmembrane regions and part of the N-terminal sequence are similar to the orphan GPCR intimal thickness related receptor (GPR180). Furthermore, the similarity of the transmembrane domain region indicates that SR12 belongs to the rhodopsin superfamily. GPR180 is an orphan receptor that plays a role in the vascular remodeling and maintenance of vascular restenosis in mice [76] and there is evidence of its relationship with the inhibition of cAMP synthesis in cells stimulated with L-lactate [77]. Using bioinformatics, Gupta et al. (2015) identified the presence of a P-loop domain and four amino acids capable of interacting with ATP, which would define SR12 as a purinergic receptor [71], and this theory has been reinforced recently with binding experiments to ATP and the purinergic receptor-specific inhibitor prasugrel [72]. Recently, the GPCR-related features of PfSR12 were studied in depth by Pereira et al. (2020). In this work, using a heterologous expression system of PfSR12 in HEK293 mammalian cells and BRET-based biosensors, several activation tests of signaling pathways downstream to GPCRs were performed. Using thrombin as a possible agonist of PfSR12, an increase in intracellular calcium concentration was detected, as well as diacylglycerol production and protein kinase C activation. It is worth noting that the detection of these intermediates was dependent on the concentration of thrombin and the amount of transfected PfSR12, which is consistent with a GPCR activation mediated by Gq/11 family proteins. In fact, using a specific pharmacological inhibitor or knockout of the Gq/11 family causes a loss of signal, but rescuing Gq, G11, and G14 proteins restores the signal or amplifies it. However, it has not been possible so far to separate the PfSR12 signaling described here from endogenous signaling of other receptors present in HEK293 cells, such as protease-activated receptors and muscarinic receptors [73].

PfSR25 has the shortest N-terminal end among the four putative *P. falciparum* GPCRs, containing only 22 amino acids, which classifies it as a putative class A receptor as described using binary topology pattern methodology [63]. Another feature that stands out is the first extracellular loop, which is longer than in the other receptors, containing 62 amino acids. PfSR25 can be found in all *Plasmodium* species with high similarity, but there is no homologous protein in human, but for some of the phylum Apicomplexa (*Babesia bovis*, *Theileria annulata* and *Theileria parva*). Using a gene-edited *P. falciparum* strain for knocking out PfSR25, Moraes et al. (2017) described PfSR25 as a sensor capable of detecting extracellular K^+^ concentration changes common in the egress/invasion process [78], activating PLC, and increasing cytoplasmic calcium concentration from the release of intracellular stores, as expected for a Gq-mediated signaling pathway. Furthermore, PfSR25 knockout experiments related it to control cell volume after hyperosmotic stress, to decreased survival and metacaspase expression after treatment with nitric oxide donor sodium nitroprusside (SNP), antimalarials, and when subjected to starvation [74]. In a screening with compounds derived from 1,2,3-triazoles, Santos et al. (2020) identified thirty-one compounds with antimalarial activity in the micromolar range, using wild *P. falciparum* strains. After selecting these compounds, they were tested in a knockout strain for PfSR25, confirming the results found previously: the wild-type strain shows a higher IC50 for antimalarials than the PfSR25 knockout strain [66]. Following the same approach, antimalarials commonly used to treat the disease were tested, along with selected compounds from the Medicine for Malaria Venture known to inhibit invasion and/or egress processes. Using flow cytometry, it was observed that lumefantrine and piperaquine are more active in parasites lacking PfSR25, while other antimalarials such as artemisinin, mefloquine, primaquine, and pyrimethamine showed no differences, indicating a role for PfSR25 in a mechanism of action differentiated so far for piperaquine and lumefantrine [75].

## 4. Conclusions

The importance of GPCRs for the organization of eukaryotes is evident, and researchers are indicating the new roles and functions of these receptors for the regulation of cell signaling pathways, such as G-protein-independent signaling, endosomal signaling, and biased signaling. The trend is that GPCRs will continue to be prime candidates as treatment targets for diseases affecting humans. The presence of GPCRs in intracellular parasites like *Plasmodium*, would indicate a divergent evolutionary branch from the one that gave rise to the GPCRs known in humans. Putting together the uniqueness of putative GPCRs in *Plasmodium* and their function in signaling in these parasites, a potentially promising new class of antimalarial drugs may emerge with GPCRs as targets.

The evidence for GPCR-like mediated signaling in *Plasmodium* goes far beyond the presence of several genes and/or candidates for the canonical components of this pathway (as shown in Table 1). There are several studies reporting the activation of kinases and the production or inhibition of cAMP production from the treatment of *Plasmodium* cells with various compounds. Worth noting is the PLC/IP3 signaling pathway, which is classically mediated by Gαq/11 in mammals. Despite unsuccessful attempts to identify the IP3 receptor, the remainder of the pathway has been shown to be functional, with cleavage of membrane phosphoinositols, IP3 release, and calcium release from the endoplasmic reticulum. This evidence leads us to believe that there are proteins that perform the same function in *Plasmodium* still unknown and quite divergent from homologs in humans.

The four main candidates for GPCRs have increasingly attracted the attention of malaria researchers. PfSR10, PfSR12 and PfSR25 are currently the best candidates with the most advanced studies: it is possible to infer functions in cell signaling and possible relationships with ligands, and these two candidates are the only ones with the common domain of LUSTR GPCRs. Apparently PfSR1 is very divergent from any other GPCR, lacking any conserved domain characteristic of that family, only a similarity to a protein that probably has a structural role (CLPTM1). PfSR25 is a promising candidate due to its relationship with the PLC/IP3 signaling pathway. In addition to the four putative receptors, we should highlight the putative integral membrane protein GPR180 (PF3D7_1213500), which despite having a localization signal to the apicoplast rather than the plasma membrane, the similarities to the orphan GPCR GPR180 are evident.

The presence of secondary messengers and signaling machinery (as shown for Sr10 and Sr25), the partially conserved structure of the transmembrane regions (as shown for Sr10 and SR12), and the possibility of G-protein-independent signaling and possible dimerization with other GPCRs (as shown for Sr12) are evidence of the presence of GPCRs in *Plasmodium*. Further studies need to be performed in order to elucidate the three-dimensional structure for more accurate comparison with known GPCRs, including ligand design and identification. In addition, possible intermediates between putative receptors and secondary messengers need to be identified, aiming to elucidate a G-protein homologue in this organism.

## Figures and Tables

**Table 1:** Common representatives of a canonical GPCR signaling pathway in *H. sapiens* and *P. falciparum*.

Protein	*H. sapiens* ^1^	*P. falciparum*
GRK	AAA58620; AAA60175	-
Gα	AAC50363; NP_002064; AAH87537	- ^2^
Gβ	AAA35922	-
Gγ	AAC39869; AAK53385	-
Adenylate cyclase	CAA84552	PF3D7_1404600
Guanylyl cyclase	NP_000848	PF3D7_1360500
Phosphodiesterase	AAA03592	PF3D7_1321500
Phospholipase C	AAA60112	PF3D7_1013500
PKC	EAW89014	-
PKA	AAC41690	PF3D7_0934800
β-Arrestin	AAH03636; AAH67368	-
Clathrin	NP_009029; NP_001825	PF3D7_1435500; PF3D7_1219100
MAPK	NP_002736	PF3D7_1113900
EPAC	NP_001362802	PF3D7_1417400
IP3 receptor	NP_002215	-

^1^ Due to the abundance of isoforms and subunits present for each protein cited in this organism, a reduced number of examples are given for each component in this table. ^2^ Multiple proteins with no defined function have conserved domains referring to this component of the signaling pathway.

**Table 2:** Characteristics of GPCR-like proteins found in *Plasmodium* genus.

	SR1	SR10	SR12	SR25
**SIZE (AA)**	773	655	470	357
**N TERMINAL LENGTH**	508	381	232	51
**C TERMINAL LENGTH**	23	35	18	9
**PREDICTED** **CLEAVAGE SITE**	-	30–31 (SNG-QL)	21–22 (YYL-TK)	28–29 (VFT-AF)
**7 TRANSMEMBRANEREGION**	509–750	382–620	233–452	217–440
**PREDICTED** **CLASSIFICATION**	Class C	Class A	-	Class A
**SIGNAL PEPTIDE** **(SIGNALP 3.0)**	no	yes	yes	yes
**SIGNAL PEPTIDE** **(SIGNALP 5.0)**	no	no	no	no
**SIGNAL PEPTIDE** **(PHOBIUS)**	no	no ^1^	no ^1^	yes
**TRANSMEMBRANE** **DOMAINS**	7	8 ^2^	7	8 ^2^
**EXPRESSED MAINLY IN (STAGES) ^3^**	Schizont, gametocytes, ookinete	Ring, schizont, gametocytes	Trophozoite, schizont, gametocyte, ookinete	Ring, trophozoite, schizont, gametocyte
**FOUND IN (SPECIES) ^3,4^**	*P. falciparum, P. reichenowi*	*P. falciparum, P. reichenowi, P. berghei, P. chabaudi, P. gallinaceum, P. knowlesi, P. malariae, P. ovale, P. vivax*	*P. falciparum, P. berghei, P. chabaudi, P. cynomolgi, P. gallinaceum, P. knowlesi, P. malariae, P. ovale, P. reichenowi, P. vivax, P. yoelii*	*P. falciparum, P. berghei, P. gallinaceum, P. knowlesi, P. malariae, P. ovale, P. reichenowi, P. vivax*
**KO PHENOTYPE**	Slow growth rate	Slow growth rate. Reduced cycle length.	Dispensable	Essential [65]/dispensable [66]
**REFERENCES**	-	[67,68,69,70]	[71,72,73]	[66,74,75]

^1^ Approximately 45% probability of signal peptide. ^2^ 1 N-terminal transmembrane (signal peptide) + 7 transmembrane cluster. ^3^ As described in PlasmoDB. ^4^ High similarity or same annotation.

## References

[1] Lander E.S., Linton L.M., Birren B., Nusbaum C., Zody M.C., Baldwin J., Devon K., Dewar K., Doyle M., Fitzhugh W. (2001). Initial sequencing and analysis of the human genome. Nature.

[2] Mombaerts P. (2004). Genes and ligands for odorant, vomeronasal and taste receptors. Nat. Rev. Neurosci..

[3] Overington J.P., Al-Lazikani B., Hopkins A. (2006). How many drug targets are there?. Nat. Rev. Drug Discov..

[4] Rask-Andersen M., Masuram S., Schiöth H.B. (2013). The Druggable Genome: Evaluation of Drug Targets in Clinical Trials Suggests Major Shifts in Molecular Class and Indication. Annu. Rev. Pharmacol. Toxicol..

[5] Manglik A., Kobilka B. (2014). The role of protein dynamics in GPCR function: Insights from the β2AR and rhodopsin. Curr. Opin. Cell Biol..

[6] Latorraca N.R., Venkatakrishnan A.J., Dror R.O. (2016). GPCR Dynamics: Structures in Motion. Chem. Rev..

[7] Ahmad R., Lahuna O., Sidibe A., Daulat A., Zhang Q., Luka M., Guillaume J.-L., Gallet S., Guillonneau F., Hamroune J. (2020). GPR50-Ctail cleavage and nuclear translocation: A new signal transduction mode for G protein-coupled receptors. Cell. Mol. Life Sci..

[8] Venkatakrishnan A.J., Deupi X., Lebon G., Heydenreich F., Flock T., Miljus T., Balaji S., Bouvier M., Veprintsev D., Tate C.G. (2016). Diverse activation pathways in class A GPCRs converge near the G-protein-coupling region. Nature.

[9] Kenakin T. (2013). New concepts in pharmacological efficacy at 7TM receptors: IUPHAR Review 2. Br. J. Pharmacol..

[10] Rosenbaum D.M., Rasmussen S.G.F., Kobilka B.K. (2009). The structure and function of G-protein-coupled receptors. Nature.

[11] Mizuno T.M., Makimura H., Mobbs C.V. (2003). The physiological function of the agouti-related peptide gene: The control of weight and metabolic rate. Ann. Med..

[12] Downes G., Gautam N. (1999). The G Protein Subunit Gene Families. Genomics.

[13] Lokits A.D., Indrischek H., Meiler J., Hamm H.E., Stadler P.F. (2018). Tracing the evolution of the heterotrimeric G protein α subunit in Metazoa. BMC Evol. Biol..

[14] Hilger D., Masureel M., Kobilka B.K. (2018). Structure and dynamics of GPCR signaling complexes. Nat. Struct. Mol. Biol..

[15] Kristiansen K. (2004). Molecular mechanisms of ligand binding, signaling, and regulation within the superfamily of G-protein-coupled receptors: Molecular modeling and mutagenesis approaches to receptor structure and function. Pharmacol. Ther..

[16] Oldham W., Hamm H.E. (2008). Heterotrimeric G protein activation by G-protein-coupled receptors. Nat. Rev. Mol. Cell Biol..

[17] Khan S.M., Sleno R., Gora S., Zylbergold P., Laverdure J.-P., Labbé J.-C., Miller G.J., Hébert T.E. (2013). The Expanding Roles of Gβγ Subunits in G Protein–Coupled Receptor Signaling and Drug Action. Pharmacol. Rev..

[18] Gurevich E.V., Tesmer J., Mushegian A., Gurevich V. (2012). G protein-coupled receptor kinases: More than just kinases and not only for GPCRs. Pharmacol. Ther..

[19] Liang Y.-L., Khoshouei M., Radjainia M., Zhang Y., Glukhova A., Tarrasch J., Thal D., Furness S., Christopoulos G., Coudrat T. (2017). Phase-plate cryo-EM structure of a class B GPCR–G-protein complex. Nature.

[20] Zhang Y., Sun B., Feng D., Hu H., Chu M., Qu Q., Tarrasch J.T., Li S., Kobilka T.S., Kobilka B.K. (2017). Cryo-EM structure of the activated GLP-1 receptor in complex with a G protein. Nature.

[21] Zhou X.E., He Y., de Waal P., Gao X., Kang Y., Van Eps N., Yin Y., Pal K., Goswami D., White T.A. (2017). Identification of Phosphorylation Codes for Arrestin Recruitment by G Protein-Coupled Receptors. Cell.

[22] Cahill T.J., Thomsen A., Tarrasch J.T., Plouffe B., Nguyen A., Yang F., Huang L.-Y., Kahsai A.W., Bassoni D.L., Gavino B.J. (2017). Distinct conformations of GPCR–β-arrestin complexes mediate desensitization, signaling, and endocytosis. Proc. Natl. Acad. Sci. USA.

[23] Kumari P., Srivastava A., Ghosh E., Ranjan R., Dogra S., Yadav P.N., Shukla A.K. (2017). Core engagement with β-arrestin is dispensable for agonist-induced vasopressin receptor endocytosis and ERK activation. Mol. Biol. Cell.

[24] Moaven H., Koike Y., Jao C.C., Gurevich V., Langen R., Chen J. (2013). Visual arrestin interaction with clathrin adaptor AP-2 regulates photoreceptor survival in the vertebrate retina. Proc. Natl. Acad. Sci. USA.

[25] Ménard R., Tavares J., Cockburn I., Markus M., Zavala F., Amino R. (2013). Looking under the skin: The first steps in malarial infection and immunity. Nat. Rev. Genet..

[26] Tavares J., Formaglio P., Thiberge S., Mordelet E., Van Rooijen N., Medvinsky A., Ménard R., Amino R. (2013). Role of host cell traversal by the malaria sporozoite during liver infection. J. Exp. Med..

[27] Prudêncio M., Rodriguez A., Mota M.M. (2006). The silent path to thousands of merozoites: The Plasmodium liver stage. Nat. Rev. Genet..

[28] Grüring C., Heiber A., Kruse F., Ungefehr J., Gilberger T.-W., Spielmann T. (2011). Development and host cell modifications of *Plasmodium falciparum* blood stages in four dimensions. Nat. Commun..

[29] Garcia C.R.S., Markus R., Madeira L. (2001). Tertian and Quartan Fevers: Temporal Regulation in Malarial Infection. J. Biol. Rhythm..

[30] Bansal A., Molina-Cruz A., Brzostowski J., Liu P., Luo Y., Gunalan K., Li Y., Ribeiro J.M.C., Miller L.H. (2018). PfCDPK1 is critical for malaria parasite gametogenesis and mosquito infection. Proc. Natl. Acad. Sci. USA.

[31] Graumans W., Tadesse F.G., Andolina C., Van Gemert G.-J., Teelen K., Lanke K., Gadisa E., Yewhalaw D., Van De Vegte-Bolmer M., Siebelink-Stoter R. (2017). Semi-high-throughput detection of *Plasmodium falciparum* and *Plasmodium vivax* oocysts in mosquitoes using bead-beating followed by circumsporozoite ELISA and quantitative PCR. Malar. J..

[32] Koyama F.C., Chakrabarti D., Garcia C.R. (2009). Molecular machinery of signal transduction and cell cycle regulation in Plasmodium. Mol. Biochem. Parasitol..

[33] Hotta C., Gazarini M., Beraldo F.H., Varotti F.D.P., Lopes C., Markus R., Pozzan T., Garcia C. (2000). Calcium-dependent modulation by melatonin of the circadian rhythm in malarial parasites. Nature.

[34] Beraldo F.H., Garcia C.R.S. (2005). Products of tryptophan catabolism induce Ca^2+^ release and modulate the cell cycle of *Plasmodium falciparum* malaria parasites. J. Pineal Res..

[35] Budu A., Peres R., Bueno V.B., Catalani L.H., Garcia C.R.D.S. (2006). N1-acetyl-N2-formyl-5-methoxykynuramine modulates the cell cycle of malaria parasites. J. Pineal Res..

[36] Lima W.R., Tessarin-Almeida G., Rozanski A., Parreira K.S., Moraes M.S., Martins D.C., Hashimoto R., Galante P., Garcia C.R. (2016). Signaling transcript profile of the asexual intraerythrocytic development cycle of *Plasmodium falciparum* induced by melatonin and cAMP. Genes Cancer.

[37] Koyama F.C., Azevedo M.F., Budu A., Chakrabarti D., Garcia C.R.S. (2014). Melatonin-Induced Temporal up-Regulation of Gene Expression Related to Ubiquitin/Proteasome System (UPS) in the Human Malaria Parasite *Plasmodium falciparum*. Int. J. Mol. Sci..

[38] Lima W.R., Moraes M., Alves E., Azevedo M., Passos D.O., Garcia C.R.S. (2012). The PfNF-YB transcription factor is a downstream target of melatonin and cAMP signalling in the human malaria parasite *Plasmodium falciparum*. J. Pineal Res..

[39] Lima W.R., Martins D.C., Parreira K.S., Scarpelli P., De Moraes M.S., Topalis P., Hashimoto R., Garcia C.R.S. (2017). Genome-wide analysis of the human malaria parasite *Plasmodium falciparum* transcription factor PfNF-YB shows interaction with a CCAAT motif. Oncotarget.

[40] Singh M.K., Tessarin-Almeida G., Dias B.K.M., Pereira P.S., Costa F., Przyborski J.M., Garcia C.R.S. (2021). A nuclear protein, PfMORC confers melatonin dependent synchrony of the human malaria parasite *P. falciparum* in the asexual stage. Sci. Rep..

[41] Dorin D., Semblat J.-P., Poullet P., Alano P., Goldring J.P.D., Whittle C., Patterson S., Chakrabarti D., Doerig C. (2005). PfPK7, an atypical MEK-related protein kinase, reflects the absence of classical three-component MAPK pathways in the human malaria parasite *Plasmodium falciparum*. Mol. Microbiol..

[42] Dorin-Semblat D., Sicard A., Doerig C., Ranford-Cartwright L., Doerig C. (2008). Disruption of the Pf PK7 Gene Impairs Schizogony and Sporogony in the Human Malaria Parasite *Plasmodium falciparum*. Eukaryot. Cell.

[43] Koyama F., Ribeiro R., Garcia J., Mauro F., Chakrabarti D., Garcia C. (2013). Ubiquitin Proteassome System and the atypical kinase PfPK7 are involved in melatonin signaling in *Plasmodium falciparum*. J. Pineal Res..

[44] Pease B.N., Huttlin E., Jedrychowski M.P., Dorin-Semblat D., Sebastiani D., Segarra D.T., Roberts B.F., Chakrabarti R., Doerig C., Gygi S.P. (2018). Characterization of *Plasmodium falciparum* Atypical Kinase PfPK7–Dependent Phosphoproteome. J. Proteome Res..

[45] Dias B.K., Nakabashi M., Alves M.R.R., Portella D.P., Santos B.M., Almeida F.C.D.S., Ribeiro R.Y., Schuck D.C., Jordão A.K., Garcia C.R. (2020). The *Plasmodium falciparum* eIK1 kinase (PfeIK1) is central for melatonin synchronization in the human malaria parasite. Melatotosil blocks melatonin action on parasite cell cycle. J. Pineal Res..

[46] Alves E., Bartlett P.J., Garcia C.R., Thomas A.P. (2011). Melatonin and IP3-induced Ca^2+^ Release from Intracellular Stores in the Malaria Parasite *Plasmodium falciparum* within Infected Red Blood Cells. J. Biol. Chem..

[47] Pecenin M.F., Borges-Pereira L., Levano-Garcia J., Budu A., Alves E., Mikoshiba K., Thomas A., Garcia C.R. (2018). Blocking IP 3 signal transduction pathways inhibits melatonin-induced Ca^2+^ signals and impairs *P. falciparum* development and proliferation in erythrocytes. Cell Calcium.

[48] Beraldo F.H., Mikoshiba K., Garcia C.R.S. (2007). Human malarial parasite, *Plasmodium falciparum*, displays capacitative calcium entry: 2-aminoethyl diphenylborinate blocks the signal transduction pathway of melatonin action on the *P. falciparum* cell cycle. J. Pineal Res..

[49] Garcia C.R., Alves E., Pereira P.H.S., Bartlett P.J., Thomas A.P., Mikoshiba K., Plattner H., Sibley L.D. (2017). InsP3 Signaling in Apicomplexan Parasites. Curr. Top. Med. Chem..

[50] Alves E., Nakaya H., Guimarães E., Garcia C.R.S. (2021). Combining IP affinity chromatography and bioinformatics reveals a novel protein-IP_3_ binding site on *Plasmodium falciparum* MDR1 transporter. bioRxiv.

[51] Cruz L.N., Juliano M.A., Budu A., Juliano L., Holder A.A., Blackman M.J., Garcia C.R. (2012). Extracellular ATP triggers proteolysis and cytosolic Ca^2+^ rise in *Plasmodium berghei* and *Plasmodium yoelii* malaria parasites. Malar. J..

[52] Levano-Garcia J., Dluzewski A.R., Markus R.P., Garcia C.R.S. (2010). Purinergic signalling is involved in the malaria parasite *Plasmodium falciparum* invasion to red blood cells. Purinergic Signal..

[53] Budu A., Garcia C.R. (2012). Generation of second messengers in Plasmodium. Microbes Infect..

[54] Baker D.A., Kelly J.M. (2004). Purine nucleotide cyclases in the malaria parasite. Trends Parasitol..

[55] Weber J.H., Vishnyakov A., Hambach K., Schultz A., Schultz J.E., Linder J.U. (2003). Adenylyl cyclases from Plasmodium, Paramecium and Tetrahymena are novel ion channel/enzyme fusion proteins. Cell. Signal..

[56] Ono T., Cabrita-Santos L., Leitao R., Bettiol E., Purcell L.A., Diaz-Pulido O., Andrews L.B., Tadakuma T., Bhanot P., Mota M.M. (2008). Adenylyl Cyclase α and cAMP Signaling Mediate Plasmodium Sporozoite Apical Regulated Exocytosis and Hepatocyte Infection. PLoS Pathog..

[57] Singh S., Alam M.M., Pal-Bhowmick I., Brzostowski J.A., Chitnis C.E. (2010). Distinct External Signals Trigger Sequential Release of Apical Organelles during Erythrocyte Invasion by Malaria Parasites. PLoS Pathog..

[58] Dawn A., Singh S., More K.R., Siddiqui F.A., Pachikara N., Ramdani G., Langsley G., Chitnis C.E. (2014). The Central Role of cAMP in Regulating *Plasmodium falciparum* Merozoite Invasion of Human Erythrocytes. PLoS Pathog..

[59] Thélu J., Bracchi V., Burnod J., Ambroise-Thomas P. (1994). Evidence for expression of a Ras-like and a stage specific GTP binding homologous protein by *Plasmodium falciparum*. Cell. Signal..

[60] Dyer M., Day K. (2000). Expression of *Plasmodium falciparum* trimeric G proteins and their involvement in switching to sexual development. Mol. Biochem. Parasitol..

[61] Kaiser A., Langer B., Przyborski J., Kersting D., Krüger M. (2015). A Putative Non-Canonical Ras-Like GTPase from *P. falciparum*: Chemical Properties and Characterization of the Protein. PLoS ONE.

[62] Harrison T., Samuel B.U., Akompong T., Hamm H., Mohandas N., Lomasney J.W., Haldar K. (2003). Erythrocyte G Protein-Coupled Receptor Signaling in Malarial Infection. Science.

[63] Inoue Y., Ikeda M., Shimizu T. (2004). Proteome-wide classification and identification of mammalian-type GPCRs by binary topology pattern. Comput. Biol. Chem..

[64] Madeira L., Galante P.A.F., Budu A., Azevedo M.F., Malnic B., Garcia C.R.S. (2008). Genome-Wide Detection of Serpentine Receptor-Like Proteins in Malaria Parasites. PLoS ONE.

[65] Zhang M., Wang C., Otto T.D., Oberstaller J., Liao X., Adapa S.R., Udenze K., Bronner I.F., Casandra D., Mayho M. (2018). Uncovering the essential genes of the human malaria parasite *Plasmodium falciparum* by saturation mutagenesis. Science.

[66] dos Santos B.M., Gonzaga D.T.G., da Silva F.C., Ferreira V.F., Garcia C.R.S. (2020). *Plasmodium falciparum* Knockout for the GPCR-Like PfSR25 Receptor Displays Greater Susceptibility to 1,2,3-Triazole Compounds That Block Malaria Parasite Development. Biomolecules.

[67] Tchoufack E.J.N., Hahnfeld L., Pitschelatow G., Bennink S., Pradel G. (2020). The endoplasmic reticulum-resident serpentine receptor SR10 has important functions for asexual and sexual blood stage development of *Plasmodium falciparum*. Mol. Biochem. Parasitol..

[68] Marapana D.S., Dagley L.F., Sandow J.J., Nebl T., Triglia T., Pasternak M., Dickerman B.K., Crabb B.S., Gilson P.R., Webb A.I. (2018). Plasmepsin V cleaves malaria effector proteins in a distinct endoplasmic reticulum translocation interactome for export to the erythrocyte. Nat. Microbiol..

[69] Aurrecoechea C., Brestelli J., Brunk B.P., Dommer J., Fischer S., Gajria B., Gao X., Gingle A., Grant G., Harb O.S. (2009). PlasmoDB: A functional genomic database for malaria parasites. Nucleic Acids Res..

[70] Subudhi A.K., O’Donnell A.J., Ramaprasad A., Abkallo H.M., Kaushik A., Ansari H.R., Abdel-Haleem A., Ben Rached F., Kaneko O., Culleton R. (2020). Malaria parasites regulate intra-erythrocytic development duration via serpentine receptor 10 to coordinate with host rhythms. Nat. Commun..

[71] Gupta S., Singh D., Singh S. (2015). In silico characterization of *Plasmodium falciparum* purinergic receptor: A novel chemotherapeutic target. Syst. Synth. Biol..

[72] Gupta S., Joshi N., Saini M., Singh S. (2020). Antimalarial and *Plasmodium falciparum* serpentine receptor 12 targeting effect of a purinergic receptor antagonist FDA approved drug. bioRxiv.

[73] Pereira P.H.S., Brito G., Moraes M., Kiyan C.L., Avet C., Bouvier M., Garcia C.R. (2020). BRET sensors unravel that *Plasmodium falciparum* serpentine receptor 12 (PfSR12) increases surface expression of mammalian GPCRs in HEK293 cells. bioRxiv.

[74] Moraes M.S., Budu A., Singh M., Borges-Pereira L., Levano-Garcia J., Curra C., Picci L., Pace T., Ponzi M., Pozzan T. (2017). *Plasmodium falciparum* GPCR-like receptor SR25 mediates extracellular K^+^ sensing coupled to Ca^2+^ signaling and stress survival. Sci. Rep..

[75] Santos B.M., Dias B.K.M., Nakabashi M., Garcia C.R.S. (2021). The Knockout for G Protein-Coupled Receptor-Like PfSR25 Increases the Susceptibility of Malaria Parasites to the Antimalarials Lumefantrine and Piperaquine but Not to Medicine for Malaria Venture Compounds. Front. Microbiol..

[76] Tsukada S., Iwai M., Nishiu J., Itoh M., Tomoike H., Horiuchi M., Nakamura Y., Tanaka T. (2003). Inhibition of Experimental Intimal Thickening in Mice Lacking a Novel G-Protein–Coupled Receptor. Circulation.

[77] Mosienko V., Rasooli-Nejad S., Kishi K., De Both M., Jane D., Huentelman M.J., Kasparov S., Teschemacher A.G. (2018). Putative Receptors Underpinning l-Lactate Signalling in Locus Coeruleus. Neuroglia.

[78] Kumar K.A., Garcia C., Chandran V.R., Van Rooijen N., Zhou Y., Winzeler E., Nussenzweig V. (2007). Exposure of Plasmodium sporozoites to the intracellular concentration of potassium enhances infectivity and reduces cell passage activity. Mol. Biochem. Parasitol..

